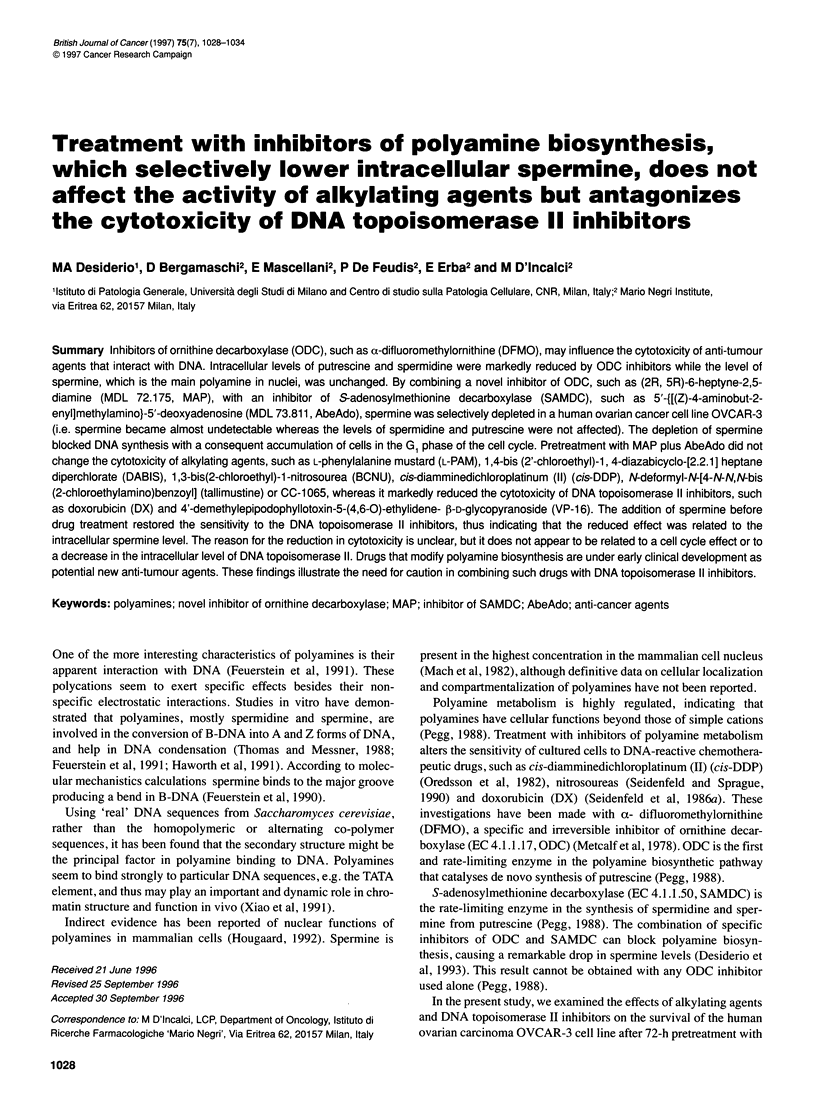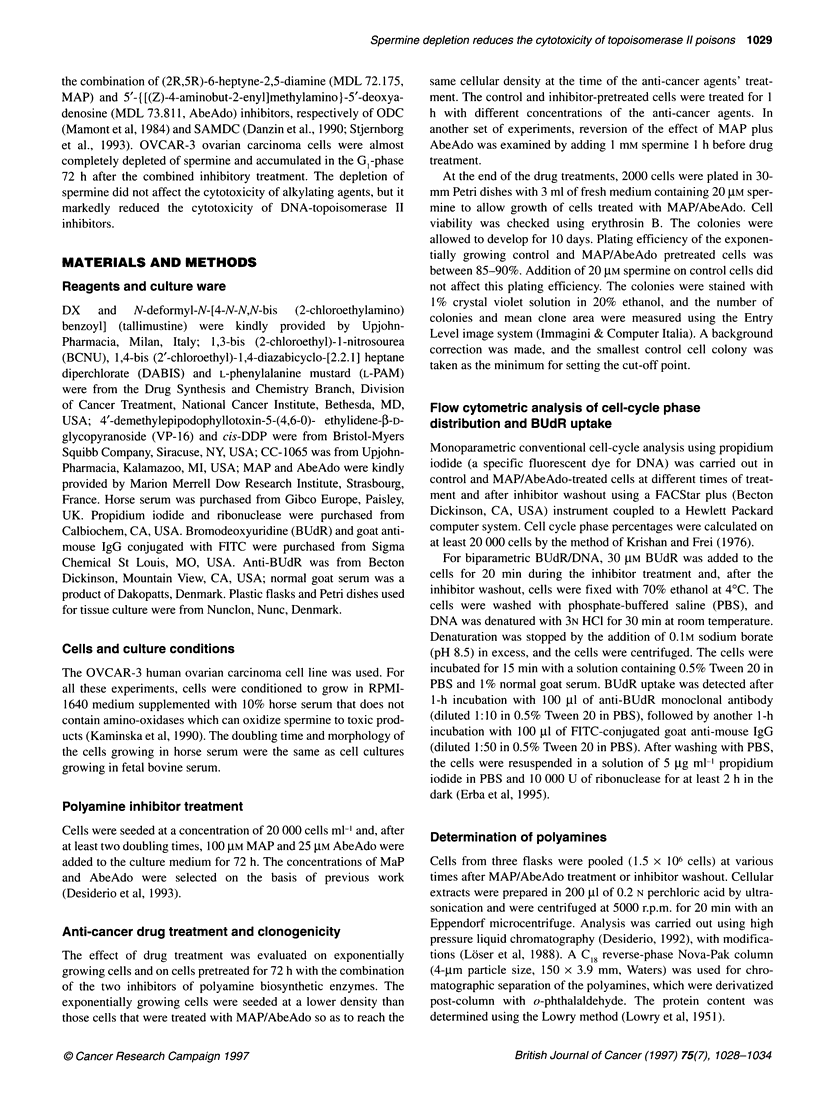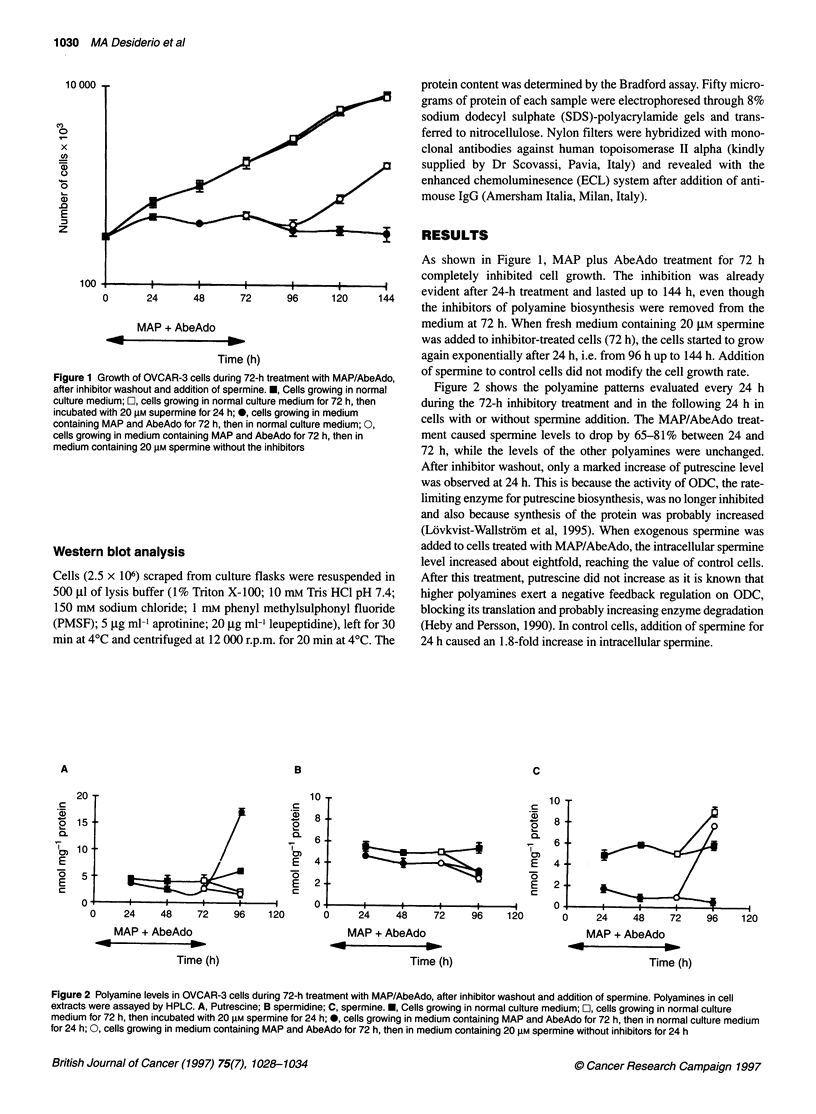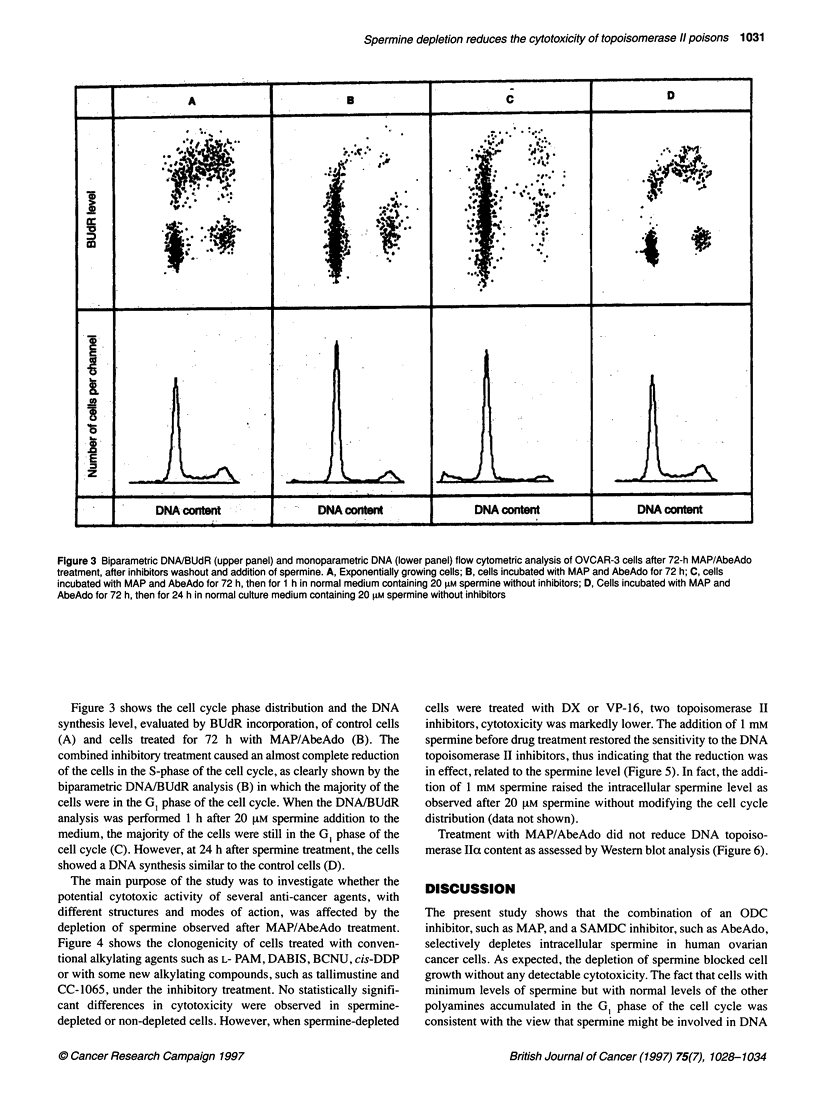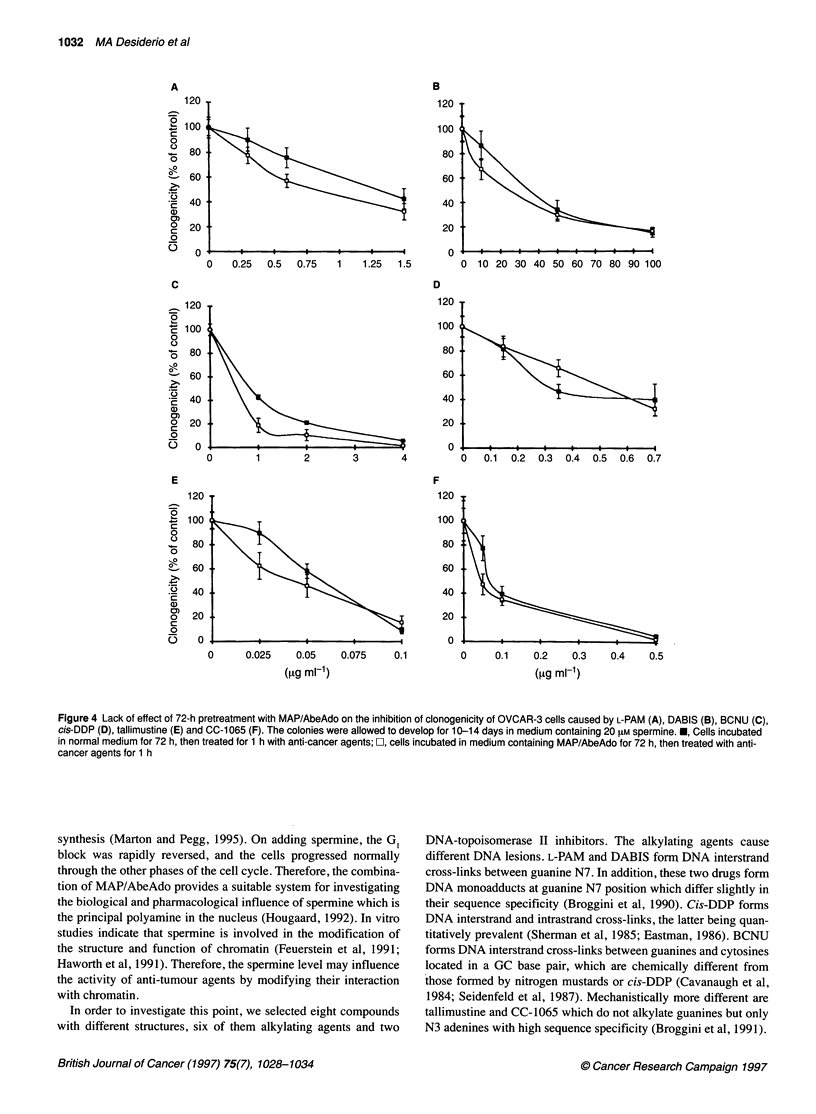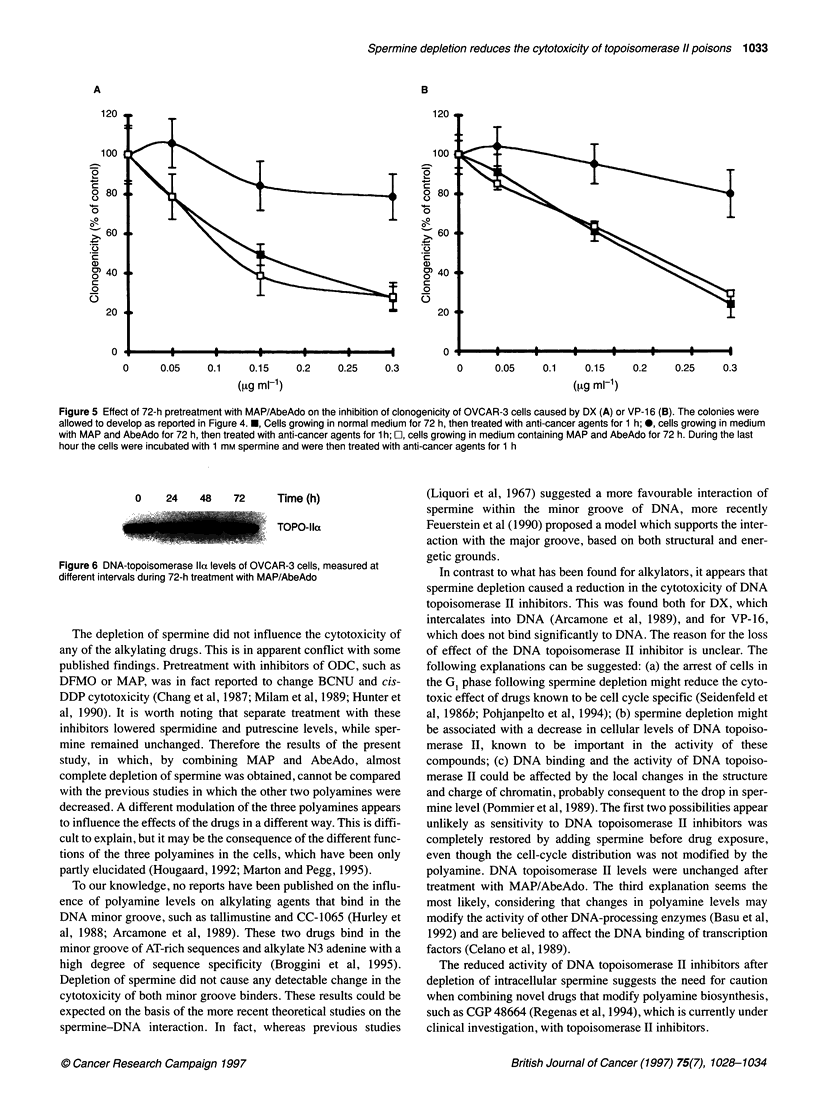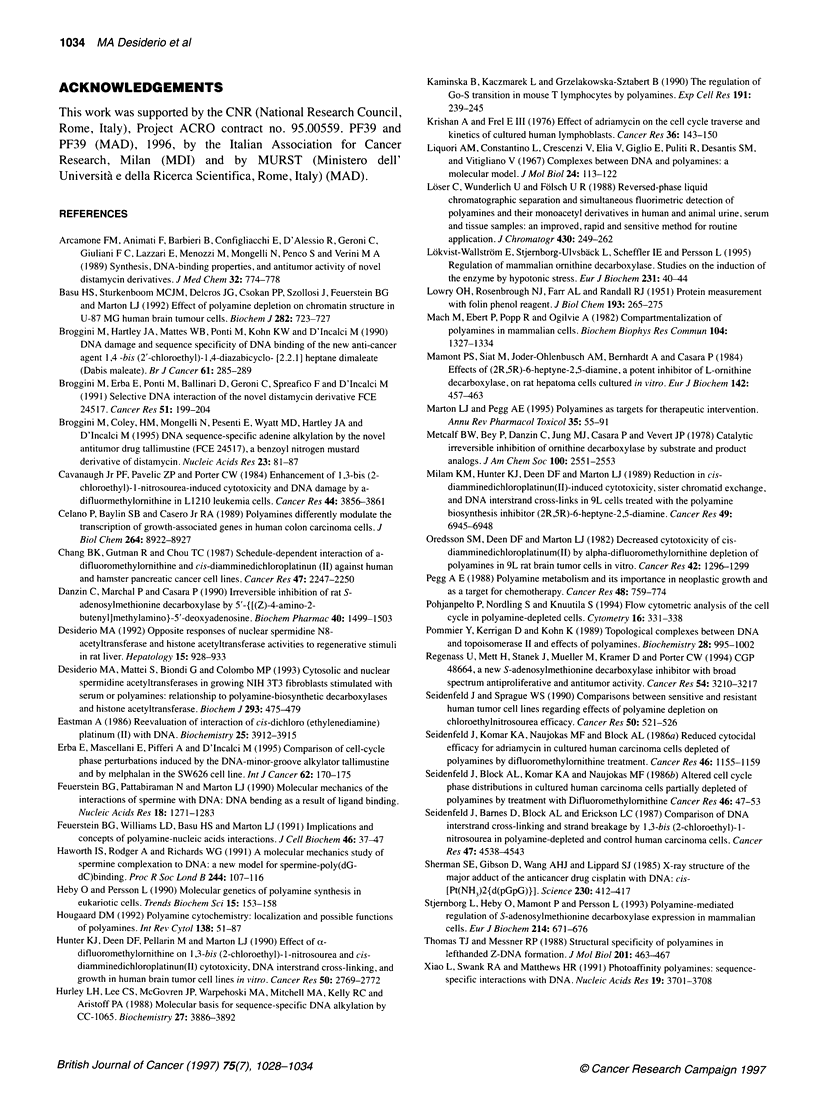# Treatment with inhibitors of polyamine biosynthesis, which selectively lower intracellular spermine, does not affect the activity of alkylating agents but antagonizes the cytotoxicity of DNA topoisomerase II inhibitors.

**DOI:** 10.1038/bjc.1997.176

**Published:** 1997

**Authors:** M. A. Desiderio, D. Bergamaschi, E. Mascellani, P. De Feudis, E. Erba, M. D'Incalci

**Affiliations:** Istituto di Patologia Generale, UniversitÃ degli Studi di Milano, CNR,Milan, Italy.

## Abstract

**Images:**


British Journal of Cancer (1997) 75(7), 1028–1034
© 1997 Cancer Research Campaign

### Treatment with inhibitors of polyamine biosynthesis, which selectively lower intracellular spermine, does not affect the activity of alkylating agents but antagonizes the cytotoxicity of DNA topoisomerase II inhibitors

**MA Desiderio[1], D Bergamaschi[2], E Mascellani[2], P De Feudis[2], E Erba[2] and M D'Incalci[2]**

[1]Istituto di Patologia Generale, Università degli Studi di Milano and Centro di studio sulla Patologia Cellulare, CNR, Milan, Italy;[2] Mario Negri Institute, via Eritrea 62, 20157 Milan, Italy

**Summary** Inhibitors of ornithine decarboxylase (ODC), such as α-difluoromethylornithine (DFMO), may influence the cytotoxicity of anti-tumour agents that interact with DNA. Intracellular levels of putrescine and spermidine were markedly reduced by ODC inhibitors while the level of spermine, which is the main polyamine in nuclei, was unchanged. By combining a novel inhibitor of ODC, such as (2R, 5R)-6-heptyne-2,5-diamine (MDL 72.175, MAP), with an inhibitor of *S*-adenosylmethionine decarboxylase (SAMDC), such as 5′-{[(Z)-4-aminobut-2-enyl]methylamino}-5′-deoxyadenosine (MDL 73.811, AbeAdo), spermine was selectively depleted in a human ovarian cancer cell line OVCAR-3 (i.e. spermine became almost undetectable whereas the levels of spermidine and putrescine were not affected). The depletion of spermine blocked DNA synthesis with a consequent accumulation of cells in the $G_1$ phase of the cell cycle. Pretreatment with MAP plus AbeAdo did not change the cytotoxicity of alkylating agents, such as L-phenylalanine mustard (L-PAM), 1,4-bis (2'-chloroethyl)-1, 4-diazabicyclo-[2.2.1] heptane diperchlorate (DABIS), 1,3-bis(2-chloroethyl)-1-nitrosourea (BCNU), *cis*-diamminedichloroplatinum (II) (*cis*-DDP), *N*-deformyl-*N*-[4-*N*-*N*,*N*-bis (2-chloroethylamino)benzoyl] (tallimustine) or CC-1065, whereas it markedly reduced the cytotoxicity of DNA topoisomerase II inhibitors, such as doxorubicin (DX) and 4'-demethylepipodophyllotoxin-5-(4,6-O)-ethylidene- β-D-glycopyranoside (VP-16). The addition of spermine before drug treatment restored the sensitivity to the DNA topoisomerase II inhibitors, thus indicating that the reduced effect was related to the intracellular spermine level. The reason for the reduction in cytotoxicity is unclear, but it does not appear to be related to a cell cycle effect or to a decrease in the intracellular level of DNA topoisomerase II. Drugs that modify polyamine biosynthesis are under early clinical development as potential new anti-tumour agents. These findings illustrate the need for caution in combining such drugs with DNA topoisomerase II inhibitors.

**Keywords:** polyamines; novel inhibitor of ornithine decarboxylase; MAP; inhibitor of SAMDC; AbeAdo; anti-cancer agents

One of the more interesting characteristics of polyamines is their apparent interaction with DNA (Feuerstein et al, 1991). These polycations seem to exert specific effects besides their non-specific electrostatic interactions. Studies in vitro have demonstrated that polyamines, mostly spermidine and spermine, are involved in the conversion of B-DNA into A and Z forms of DNA, and help in DNA condensation (Thomas and Messner, 1988; Feuerstein et al, 1991; Haworth et al, 1991). According to molecular mechanistics calculations spermine binds to the major groove producing a bend in B-DNA (Feuerstein et al, 1990).

Using 'real' DNA sequences from *Saccharomyces cerevisiae*, rather than the homopolymeric or alternating co-polymer sequences, it has been found that the secondary structure might be the principal factor in polyamine binding to DNA. Polyamines seem to bind strongly to particular DNA sequences, e.g. the TATA element, and thus may play an important and dynamic role in chromatin structure and function in vivo (Xiao et al, 1991).

Indirect evidence has been reported of nuclear functions of polyamines in mammalian cells (Hougaard, 1992). Spermine is present in the highest concentration in the mammalian cell nucleus (Mach et al, 1982), although definitive data on cellular localization and compartmentalization of polyamines have not been reported.

Polyamine metabolism is highly regulated, indicating that polyamines have cellular functions beyond those of simple cations (Pegg, 1988). Treatment with inhibitors of polyamine metabolism alters the sensitivity of cultured cells to DNA-reactive chemotherapeutic drugs, such as *cis*-diamminedichloroplatinum (II) (*cis*-DDP) (Oredsson et al, 1982), nitrosoureas (Seidenfeld and Sprague, 1990) and doxorubicin (DX) (Seidenfeld et al, 1986*a*). These investigations have been made with α- difluoromethylornithine (DFMO), a specific and irreversible inhibitor of ornithine decarboxylase (EC 4.1.1.17, ODC) (Metcalf et al, 1978). ODC is the first and rate-limiting enzyme in the polyamine biosynthetic pathway that catalyses de novo synthesis of putrescine (Pegg, 1988).

*S*-adenosylmethionine decarboxylase (EC 4.1.1.50, SAMDC) is the rate-limiting enzyme in the synthesis of spermidine and spermine from putrescine (Pegg, 1988). The combination of specific inhibitors of ODC and SAMDC can block polyamine biosynthesis, causing a remarkable drop in spermine levels (Desiderio et al, 1993). This result cannot be obtained with any ODC inhibitor used alone (Pegg, 1988).

In the present study, we examined the effects of alkylating agents and DNA topoisomerase II inhibitors on the survival of the human ovarian carcinoma OVCAR-3 cell line after 72-h pretreatment with

*Received 21 June 1996*
*Revised 25 September 1996*
*Accepted 30 September 1996*

*Correspondence to:* M D'Incalci, LCP, Department of Oncology, Istituto di Ricerche Farmacologiche 'Mario Negri', Via Eritrea 62, 20157 Milan, Italy the combination of (2R,5R)-6-heptyne-2,5-diamine (MDL 72.175, MAP) and 5′-{[(Z)-4-aminobut-2-enyl]methylamino}-5′-deoxyadenosine (MDL 73.811, AbeAdo) inhibitors, respectively of ODC (Mamont et al, 1984) and SAMDC (Danzin et al., 1990; Stjernborg et al., 1993). OVCAR-3 ovarian carcinoma cells were almost completely depleted of spermine and accumulated in the $G_1$-phase 72 h after the combined inhibitory treatment. The depletion of spermine did not affect the cytotoxicity of alkylating agents, but it markedly reduced the cytotoxicity of DNA-topoisomerase II inhibitors.

### MATERIALS AND METHODS

### Reagents and culture ware

DX and *N*-deformyl-*N*-[4-*N*-*N*,*N*-bis (2-chloroethylamino) benzoyl] (tallimustine) were kindly provided by Upjohn-Pharmacia, Milan, Italy; 1,3-bis (2-chloroethyl)-1-nitrosourea (BCNU), 1,4-bis (2′-chloroethyl)-1,4-diazabicyclo-[2.2.1] heptane diperchlorate (DABIS) and L-phenylalanine mustard (L-PAM) were from the Drug Synthesis and Chemistry Branch, Division of Cancer Treatment, National Cancer Institute, Bethesda, MD, USA; 4′-demethylepipodophyllotoxin-5-(4,6-0)- ethylidene-β-D-glycopyranoside (VP-16) and *cis*-DDP were from Bristol-Myers Squibb Company, Siracuse, NY, USA; CC-1065 was from Upjohn-Pharmacia, Kalamazoo, MI, USA; MAP and AbeAdo were kindly provided by Marion Merrell Dow Research Institute, Strasbourg, France. Horse serum was purchased from Gibco Europe, Paisley, UK. Propidium iodide and ribonuclease were purchased from Calbiochem, CA, USA. Bromodeoxyuridine (BUdR) and goat anti-mouse IgG conjugated with FITC were purchased from Sigma Chemical St Louis, MO, USA. Anti-BUdR was from Becton Dickinson, Mountain View, CA, USA; normal goat serum was a product of Dakopatts, Denmark. Plastic flasks and Petri dishes used for tissue culture were from Nunclon, Nunc, Denmark.

### Cells and culture conditions

The OVCAR-3 human ovarian carcinoma cell line was used. For all these experiments, cells were conditioned to grow in RPMI-1640 medium supplemented with 10% horse serum that does not contain amino-oxidases which can oxidize spermine to toxic products (Kaminska et al, 1990). The doubling time and morphology of the cells growing in horse serum were the same as cell cultures growing in fetal bovine serum.

### Polyamine inhibitor treatment

Cells were seeded at a concentration of 20 000 cells $ml^{-1}$ and, after at least two doubling times, 100 μM MAP and 25 μM AbeAdo were added to the culture medium for 72 h. The concentrations of MaP and AbeAdo were selected on the basis of previous work (Desiderio et al, 1993).

### Anti-cancer drug treatment and clonogenicity

The effect of drug treatment was evaluated on exponentially growing cells and on cells pretreated for 72 h with the combination of the two inhibitors of polyamine biosynthetic enzymes. The exponentially growing cells were seeded at a lower density than those cells that were treated with MAP/AbeAdo so as to reach the same cellular density at the time of the anti-cancer agents' treatment. The control and inhibitor-pretreated cells were treated for 1 h with different concentrations of the anti-cancer agents. In another set of experiments, reversion of the effect of MAP plus AbeAdo was examined by adding 1 mM spermine 1 h before drug treatment.

At the end of the drug treatments, 2000 cells were plated in 30-mm Petri dishes with 3 ml of fresh medium containing 20 μM spermine to allow growth of cells treated with MAP/AbeAdo. Cell viability was checked using erythrosin B. The colonies were allowed to develop for 10 days. Plating efficiency of the exponentially growing control and MAP/AbeAdo pretreated cells was between 85–90%. Addition of 20 μM spermine on control cells did not affect this plating efficiency. The colonies were stained with 1% crystal violet solution in 20% ethanol, and the number of colonies and mean clone area were measured using the Entry Level image system (Immagini & Computer Italia). A background correction was made, and the smallest control cell colony was taken as the minimum for setting the cut-off point.

### Flow cytometric analysis of cell-cycle phase distribution and BUdR uptake

Monoparametric conventional cell-cycle analysis using propidium iodide (a specific fluorescent dye for DNA) was carried out in control and MAP/AbeAdo-treated cells at different times of treatment and after inhibitor washout using a FACStar plus (Becton Dickinson, CA, USA) instrument coupled to a Hewlett Packard computer system. Cell cycle phase percentages were calculated on at least 20 000 cells by the method of Krishan and Frei (1976).

For biparametric BUdR/DNA, 30 μM BUdR was added to the cells for 20 min during the inhibitor treatment and, after the inhibitor washout, cells were fixed with 70% ethanol at 4°C. The cells were washed with phosphate-buffered saline (PBS), and DNA was denatured with 3N HCl for 30 min at room temperature. Denaturation was stopped by the addition of 0.1M sodium borate (pH 8.5) in excess, and the cells were centrifuged. The cells were incubated for 15 min with a solution containing 0.5% Tween 20 in PBS and 1% normal goat serum. BUdR uptake was detected after 1-h incubation with 100 μl of anti-BUdR monoclonal antibody (diluted 1:10 in 0.5% Tween 20 in PBS), followed by another 1-h incubation with 100 μl of FITC-conjugated goat anti-mouse IgG (diluted 1:50 in 0.5% Tween 20 in PBS). After washing with PBS, the cells were resuspended in a solution of 5 μg $ml^{-1}$ propidium iodide in PBS and 10 000 U of ribonuclease for at least 2 h in the dark (Erba et al, 1995).

### Determination of polyamines

Cells from three flasks were pooled ($1.5 \times 10^6$ cells) at various times after MAP/AbeAdo treatment or inhibitor washout. Cellular extracts were prepared in 200 μl of 0.2 N perchloric acid by ultrasonication and were centrifuged at 5000 r.p.m. for 20 min with an Eppendorf microcentrifuge. Analysis was carried out using high pressure liquid chromatography (Desiderio, 1992), with modifications (Löser et al, 1988). A $C_{18}$ reverse-phase Nova-Pak column (4-μm particle size, 150 × 3.9 mm, Waters) was used for chromatographic separation of the polyamines, which were derivatized post-column with *o*-phthalaldehyde. The protein content was determined using the Lowry method (Lowry et al, 1951).

© Cancer Research Campaign 1997

**Figure 1** Growth of OVCAR-3 cells during 72-h treatment with MAP/AbeAdo, after inhibitor washout and addition of spermine. ■, Cells growing in normal culture medium; □, cells growing in normal culture medium for 72 h, then incubated with 20 μM supermine for 24 h; ●, cells growing in medium containing MAP and AbeAdo for 72 h, then in normal culture medium; ○, cells growing in medium containing MAP and AbeAdo for 72 h, then in medium containing 20 μM spermine without the inhibitors

### Western blot analysis

Cells (2.5 x $10^6$) scraped from culture flasks were resuspended in 500 μl of lysis buffer (1% Triton X-100; 10 mM Tris HCl pH 7.4; 150 mM sodium chloride; 1 mM phenyl methylsulphonyl fluoride (PMSF); 5 μg $ml^{-1}$ aprotinine; 20 μg $ml^{-1}$ leupeptidine), left for 30 min at 4°C and centrifuged at 12 000 r.p.m. for 20 min at 4°C. The protein content was determined by the Bradford assay. Fifty micrograms of protein of each sample were electrophoresed through 8% sodium dodecyl sulphate (SDS)-polyacrylamide gels and transferred to nitrocellulose. Nylon filters were hybridized with monoclonal antibodies against human topoisomerase II alpha (kindly supplied by Dr Scovassi, Pavia, Italy) and revealed with the enhanced chemoluminesence (ECL) system after addition of anti-mouse IgG (Amersham Italia, Milan, Italy).

### RESULTS

As shown in Figure 1, MAP plus AbeAdo treatment for 72 h completely inhibited cell growth. The inhibition was already evident after 24-h treatment and lasted up to 144 h, even though the inhibitors of polyamine biosynthesis were removed from the medium at 72 h. When fresh medium containing 20 μM spermine was added to inhibitor-treated cells (72 h), the cells started to grow again exponentially after 24 h, i.e. from 96 h up to 144 h. Addition of spermine to control cells did not modify the cell growth rate.

Figure 2 shows the polyamine patterns evaluated every 24 h during the 72-h inhibitory treatment and in the following 24 h in cells with or without spermine addition. The MAP/AbeAdo treatment caused spermine levels to drop by 65–81% between 24 and 72 h, while the levels of the other polyamines were unchanged. After inhibitor washout, only a marked increase of putrescine level was observed at 24 h. This is because the activity of ODC, the rate-limiting enzyme for putrescine biosynthesis, was no longer inhibited and also because synthesis of the protein was probably increased (Lövkvist-Wallström et al, 1995). When exogenous spermine was added to cells treated with MAP/AbeAdo, the intracellular spermine level increased about eightfold, reaching the value of control cells. After this treatment, putrescine did not increase as it is known that higher polyamines exert a negative feedback regulation on ODC, blocking its translation and probably increasing enzyme degradation (Heby and Persson, 1990). In control cells, addition of spermine for 24 h caused an 1.8-fold increase in intracellular spermine.

**Figure 2** Polyamine levels in OVCAR-3 cells during 72-h treatment with MAP/AbeAdo, after inhibitor washout and addition of spermine. Polyamines in cell extracts were assayed by HPLC. **A**, Putrescine; **B** spermidine; **C**, spermine. ■, Cells growing in normal culture medium; □, cells growing in normal culture medium for 72 h, then incubated with 20 μM spermine for 24 h; ●, cells growing in medium containing MAP and AbeAdo for 72 h, then in normal culture medium for 24 h; ○, cells growing in medium containing MAP and AbeAdo for 72 h, then in medium containing 20 μM spermine without inhibitors for 24 h

*© Cancer Research Campaign 1997*

**Figure 3** Biparametric DNA/BUdR (upper panel) and monoparametric DNA (lower panel) flow cytometric analysis of OVCAR-3 cells after 72-h MAP/AbeAdo treatment, after inhibitors washout and addition of spermine. **A**, Exponentially growing cells; **B**, cells incubated with MAP and AbeAdo for 72 h; **C**, cells incubated with MAP and AbeAdo for 72 h, then for 1 h in normal medium containing 20 μM spermine without inhibitors; **D**, Cells incubated with MAP and AbeAdo for 72 h, then for 24 h in normal culture medium containing 20 μM spermine without inhibitors

Figure 3 shows the cell cycle phase distribution and the DNA synthesis level, evaluated by BUdR incorporation, of control cells (A) and cells treated for 72 h with MAP/AbeAdo (B). The combined inhibitory treatment caused an almost complete reduction of the cells in the S-phase of the cell cycle, as clearly shown by the biparametric DNA/BUdR analysis (B) in which the majority of the cells were in the $G_1$ phase of the cell cycle. When the DNA/BUdR analysis was performed 1 h after 20 μM spermine addition to the medium, the majority of the cells were still in the $G_1$ phase of the cell cycle (C). However, at 24 h after spermine treatment, the cells showed a DNA synthesis similar to the control cells (D).

The main purpose of the study was to investigate whether the potential cytotoxic activity of several anti-cancer agents, with different structures and modes of action, was affected by the depletion of spermine observed after MAP/AbeAdo treatment. Figure 4 shows the clonogenicity of cells treated with conventional alkylating agents such as L- PAM, DABIS, BCNU, *cis*-DDP or with some new alkylating compounds, such as tallimustine and CC-1065, under the inhibitory treatment. No statistically significant differences in cytotoxicity were observed in spermine-depleted or non-depleted cells. However, when spermine-depleted cells were treated with DX or VP-16, two topoisomerase II inhibitors, cytotoxicity was markedly lower. The addition of 1 mM spermine before drug treatment restored the sensitivity to the DNA topoisomerase II inhibitors, thus indicating that the reduction was in effect, related to the spermine level (Figure 5). In fact, the addition of 1 mM spermine raised the intracellular spermine level as observed after 20 μM spermine without modifying the cell cycle distribution (data not shown).

Treatment with MAP/AbeAdo did not reduce DNA topoisomerase IIα content as assessed by Western blot analysis (Figure 6).

### DISCUSSION

The present study shows that the combination of an ODC inhibitor, such as MAP, and a SAMDC inhibitor, such as AbeAdo, selectively depletes intracellular spermine in human ovarian cancer cells. As expected, the depletion of spermine blocked cell growth without any detectable cytotoxicity. The fact that cells with minimum levels of spermine but with normal levels of the other polyamines accumulated in the $G_1$ phase of the cell cycle was consistent with the view that spermine might be involved in DNA

© Cancer Research Campaign 1997

**Figure 4** Lack of effect of 72-h pretreatment with MAP/AbeAdo on the inhibition of clonogenicity of OVCAR-3 cells caused by L-PAM (**A**), DABIS (**B**), BCNU (**C**), *cis*-DDP (**D**), tallimustine (**E**) and CC-1065 (**F**). The colonies were allowed to develop for 10–14 days in medium containing 20 μM spermine. ■, Cells incubated in normal medium for 72 h, then treated for 1 h with anti-cancer agents; □, cells incubated in medium containing MAP/AbeAdo for 72 h, then treated with anti-cancer agents for 1 h synthesis (Marton and Pegg, 1995). On adding spermine, the $G_1$ block was rapidly reversed, and the cells progressed normally through the other phases of the cell cycle. Therefore, the combination of MAP/AbeAdo provides a suitable system for investigating the biological and pharmacological influence of spermine which is the principal polyamine in the nucleus (Hougaard, 1992). In vitro studies indicate that spermine is involved in the modification of the structure and function of chromatin (Feuerstein et al, 1991; Haworth et al, 1991). Therefore, the spermine level may influence the activity of anti-tumour agents by modifying their interaction with chromatin.

In order to investigate this point, we selected eight compounds with different structures, six of them alkylating agents and two DNA-topoisomerase II inhibitors. The alkylating agents cause different DNA lesions. L-PAM and DABIS form DNA interstrand cross-links between guanine N7. In addition, these two drugs form DNA monoadducts at guanine N7 position which differ slightly in their sequence specificity (Broggini et al, 1990). *Cis*-DDP forms DNA interstrand and intrastrand cross-links, the latter being quantitatively prevalent (Sherman et al, 1985; Eastman, 1986). BCNU forms DNA interstrand cross-links between guanines and cytosines located in a GC base pair, which are chemically different from those formed by nitrogen mustards or *cis*-DDP (Cavanaugh et al, 1984; Seidenfeld et al, 1987). Mechanistically more different are tallimustine and CC-1065 which do not alkylate guanines but only N3 adenines with high sequence specificity (Broggini et al, 1991).

© Cancer Research Campaign 1997

**Figure 5** Effect of 72-h pretreatment with MAP/AbeAdo on the inhibition of clonogenicity of OVCAR-3 cells caused by DX (**A**) or VP-16 (**B**). The colonies were allowed to develop as reported in Figure 4. ■, Cells growing in normal medium for 72 h, then treated with anti-cancer agents for 1 h; ●, cells growing in medium with MAP and AbeAdo for 72 h, then treated with anti-cancer agents for 1h; □, cells growing in medium containing MAP and AbeAdo for 72 h. During the last hour the cells were incubated with 1 mM spermine and were then treated with anti-cancer agents for 1 h

**Figure 6** DNA-topoisomerase IIα levels of OVCAR-3 cells, measured at different intervals during 72-h treatment with MAP/AbeAdo

The depletion of spermine did not influence the cytotoxicity of any of the alkylating drugs. This is in apparent conflict with some published findings. Pretreatment with inhibitors of ODC, such as DFMO or MAP, was in fact reported to change BCNU and *cis*-DDP cytotoxicity (Chang et al, 1987; Milam et al, 1989; Hunter et al, 1990). It is worth noting that separate treatment with these inhibitors lowered spermidine and putrescine levels, while spermine remained unchanged. Therefore the results of the present study, in which, by combining MAP and AbeAdo, almost complete depletion of spermine was obtained, cannot be compared with the previous studies in which the other two polyamines were decreased. A different modulation of the three polyamines appears to influence the effects of the drugs in a different way. This is difficult to explain, but it may be the consequence of the different functions of the three polyamines in the cells, which have been only partly elucidated (Hougaard, 1992; Marton and Pegg, 1995).

To our knowledge, no reports have been published on the influence of polyamine levels on alkylating agents that bind in the DNA minor groove, such as tallimustine and CC-1065 (Hurley et al, 1988; Arcamone et al, 1989). These two drugs bind in the minor groove of AT-rich sequences and alkylate N3 adenine with a high degree of sequence specificity (Broggini et al, 1995). Depletion of spermine did not cause any detectable change in the cytotoxicity of both minor groove binders. These results could be expected on the basis of the more recent theoretical studies on the spermine–DNA interaction. In fact, whereas previous studies (Liquori et al, 1967) suggested a more favourable interaction of spermine within the minor groove of DNA, more recently Feuerstein et al (1990) proposed a model which supports the interaction with the major groove, based on both structural and energetic grounds.

In contrast to what has been found for alkylators, it appears that spermine depletion caused a reduction in the cytotoxicity of DNA topoisomerase II inhibitors. This was found both for DX, which intercalates into DNA (Arcamone et al, 1989), and for VP-16, which does not bind significantly to DNA. The reason for the loss of effect of the DNA topoisomerase II inhibitor is unclear. The following explanations can be suggested: (a) the arrest of cells in the $G_1$ phase following spermine depletion might reduce the cytotoxic effect of drugs known to be cell cycle specific (Seidenfeld et al, 1986*b*; Pohjanpelto et al, 1994); (b) spermine depletion might be associated with a decrease in cellular levels of DNA topoisomerase II, known to be important in the activity of these compounds; (c) DNA binding and the activity of DNA topoisomerase II could be affected by the local changes in the structure and charge of chromatin, probably consequent to the drop in spermine level (Pommier et al, 1989). The first two possibilities appear unlikely as sensitivity to DNA topoisomerase II inhibitors was completely restored by adding spermine before drug exposure, even though the cell-cycle distribution was not modified by the polyamine. DNA topoisomerase II levels were unchanged after treatment with MAP/AbeAdo. The third explanation seems the most likely, considering that changes in polyamine levels may modify the activity of other DNA-processing enzymes (Basu et al, 1992) and are believed to affect the DNA binding of transcription factors (Celano et al, 1989).

The reduced activity of DNA topoisomerase II inhibitors after depletion of intracellular spermine suggests the need for caution when combining novel drugs that modify polyamine biosynthesis, such as CGP 48664 (Regenas et al, 1994), which is currently under clinical investigation, with topoisomerase II inhibitors.

### ACKNOWLEDGEMENTS

This work was supported by the CNR (National Research Council, Rome, Italy), Project ACRO contract no. 95.00559. PF39 and PF39 (MAD), 1996, by the Italian Association for Cancer Research, Milan (MDI) and by MURST (Ministero dell' Università e della Ricerca Scientifica, Rome, Italy) (MAD).

### REFERENCES

Arcamone FM, Animati F, Barbieri B, Configliacchi E, D'Alessio R, Geroni C, Giuliani F C, Lazzari E, Menozzi M, Mongelli N, Penco S and Verini M A (1989) Synthesis, DNA-binding properties, and antitumor activity of novel distamycin derivatives. *J Med Chem* **32:** 774–778

Basu HS, Sturkenboom MCJM, Delcros JG, Csokan PP, Szollosi J, Feuerstein BG and Marton LJ (1992) Effect of polyamine depletion on chromatin structure in U-87 MG human brain tumour cells. *Biochem J* **282:** 723–727

Broggini M, Hartley JA, Mattes WB, Ponti M, Kohn KW and D'Incalci M (1990) DNA damage and sequence specificity of DNA binding of the new anti-cancer agent 1,4 -*bis* (2′-chloroethyl)-1,4-diazabicyclo- [2.2.1] heptane dimaleate (Dabis maleate). *Br J Cancer* **61:** 285–289

Broggini M, Erba E, Ponti M, Ballinari D, Geroni C, Spreafico F and D'Incalci M (1991) Selective DNA interaction of the novel distamycin derivative FCE 24517. *Cancer Res* **51:** 199–204

Broggini M, Coley, HM, Mongelli N, Pesenti E, Wyatt MD, Hartley JA and D'Incalci M (1995) DNA sequence-specific adenine alkylation by the novel antitumor drug tallimustine (FCE 24517), a benzoyl nitrogen mustard derivative of distamycin. *Nucleic Acids Res* **23:** 81–87

Cavanaugh Jr PF, Pavelic ZP and Porter CW (1984) Enhancement of 1,3-bis (2-chloroethyl)-1-nitrosourea-induced cytotoxicity and DNA damage by a-difluormethylornithine in L1210 leukemia cells. *Cancer Res* **44:** 3856–3861

Celano P, Baylin SB and Casero Jr RA (1989) Polyamines differently modulate the transcription of growth-associated genes in human colon carcinoma cells. *J Biol Chem* **264:** 8922–8927

Chang BK, Gutman R and Chou TC (1987) Schedule-dependent interaction of a-difluoromethylornithine and *cis*-diamminedichloroplatinun (II) against human and hamster pancreatic cancer cell lines. *Cancer Res* **47:** 2247–2250

Danzin C, Marchal P and Casara P (1990) Irreversible inhibition of rat *S*-adenosylmethionine decarboxylase by 5′-{[(Z)-4-amino-2-butenyl]methylamino}-5′-deoxyadenosine. *Biochem Pharmac* **40:** 1499–1503

Desiderio MA (1992) Opposite responses of nuclear spermidine N8-acetyltransferase and histone acetyltransferase activities to regenerative stimuli in rat liver. *Hepatology* **15:** 928–933

Desiderio MA, Mattei S, Biondi G and Colombo MP (1993) Cytosolic and nuclear spermidine acetyltransferases in growing NIH 3T3 fibroblasts stimulated with serum or polyamines: relationship to polyamine-biosynthetic decarboxylases and histone acetyltransferase. *Biochem J* **293:** 475–479

Eastman A (1986) Reevaluation of interaction of *cis*-dichloro (ethylenediamine) platinum (II) with DNA. *Biochemistry* **25:** 3912–3915

Erba E, Mascellani E, Pifferi A and D'Incalci M (1995) Comparison of cell-cycle phase perturbations induced by the DNA-minor-groove alkylator tallimustine and by melphalan in the SW626 cell line. *Int J Cancer* **62:** 170–175

Feuerstein BG, Pattabiraman N and Marton LJ (1990) Molecular mechanics of the interactions of spermine with DNA: DNA bending as a result of ligand binding. *Nucleic Acids Res* **18:** 1271–1283

Feuerstein BG, Williams LD, Basu HS and Marton LJ (1991) Implications and concepts of polyamine-nucleic acids interactions. *J Cell Biochem* **46:** 37–47

Haworth IS, Rodger A and Richards WG (1991) A molecular mechanics study of spermine complexation to DNA: a new model for spermine-poly(dG-dC)binding. *Proc R Soc Lond B* **244:** 107–116

Heby O and Persson L (1990) Molecular genetics of polyamine synthesis in eukariotic cells. *Trends Biochem Sci* **15:** 153–158

Hougaard DM (1992) Polyamine cytochemistry: localization and possible functions of polyamines. *Int Rev Cytol* **138:** 51–87

Hunter KJ, Deen DF, Pellarin M and Marton LJ (1990) Effect of α-difluoromethylornithine on 1,3-*bis* (2-chloroethyl)-1-nitrosourea and *cis*-diamminedichloroplatinun(II) cytotoxicity, DNA interstrand cross-linking, and growth in human brain tumor cell lines *in vitro*. *Cancer Res* **50:** 2769–2772

Hurley LH, Lee CS, McGovren JP, Warpehoski MA, Mitchell MA, Kelly RC and Aristoff PA (1988) Molecular basis for sequence-specific DNA alkylation by CC-1065. *Biochemistry* **27:** 3886–3892

Kaminska B, Kaczmarek L and Grzelakowska-Sztabert B (1990) The regulation of Go-S transition in mouse T lymphocytes by polyamines. *Exp Cell Res* **191:** 239–245

Krishan A and Frel E III (1976) Effect of adriamycin on the cell cycle traverse and kinetics of cultured human lymphoblasts. *Cancer Res* **36:** 143–150

Liquori AM, Constantino L, Crescenzi V, Elia V, Giglio E, Puliti R, Desantis SM, and Vitigliano V (1967) Complexes between DNA and polyamines: a molecular model. *J Mol Biol* **24:** 113–122

Löser C, Wunderlich U and Fölsch U R (1988) Reversed-phased liquid chromatographic separation and simultaneous fluorimetric detection of polyamines and their monoacetyl derivatives in human and animal urine, serum and tissue samples: an improved, rapid and sensitive method for routine application. *J Chromatogr* **430:** 249–262

Lökvist-Wallström E, Stjernborg-Ulvsbäck L, Scheffler IE and Persson L (1995) Regulation of mammalian ornithine decarboxylase. Studies on the induction of the enzyme by hypotonic stress. *Eur J Biochem* **231:** 40–44

Lowry OH, Rosenbrough NJ, Farr AL and Randall RJ (1951) Protein measurement with folin phenol reagent. *J Biol Chem* **193:** 265–275

Mach M, Ebert P, Popp R and Ogilvie A (1982) Compartmentalization of polyamines in mammalian cells. *Biochem Biophys Res Commun* **104:** 1327–1334

Mamont PS, Siat M, Joder-Ohlenbusch AM, Bernhardt A and Casara P (1984) Effects of (2R,5R)-6-heptyne-2,5-diamine, a potent inhibitor of L-ornithine decarboxylase, on rat hepatoma cells cultured *in vitro*. *Eur J Biochem* **142:** 457–463

Marton LJ and Pegg AE (1995) Polyamines as targets for therapeutic intervention. *Annu Rev Pharmacol Toxicol* **35:** 55–91

Metcalf BW, Bey P, Danzin C, Jung MJ, Casara P and Vevert JP (1978) Catalytic irreversible inhibition of ornithine decarboxylase by substrate and product analogs. *J Am Chem Soc* **100:** 2551–2553

Milam KM, Hunter KJ, Deen DF and Marton LJ (1989) Reduction in *cis*-diamminedichloroplatinun(II)-induced cytotoxicity, sister chromatid exchange, and DNA interstrand cross-links in 9L cells treated with the polyamine biosynthesis inhibitor (2R,5R)-6-heptyne-2,5-diamine. *Cancer Res* **49:** 6945–6948

Oredsson SM, Deen DF and Marton LJ (1982) Decreased cytotoxicity of cis-diamminedichloroplatinum(II) by alpha-difluoromethylornithine depletion of polyamines in 9L rat brain tumor cells in vitro. *Cancer Res* **42:** 1296–1299

Pegg A E (1988) Polyamine metabolism and its importance in neoplastic growth and as a target for chemotherapy. *Cancer Res* **48:** 759–774

Pohjanpelto P, Nordling S and Knuutila S (1994) Flow cytometric analysis of the cell cycle in polyamine-depleted cells. *Cytometry* **16:** 331–338

Pommier Y, Kerrigan D and Kohn K (1989) Topological complexes between DNA and topoisomerase II and effects of polyamines. *Biochemistry* **28:** 995–1002

Regenass U, Mett H, Stanek J, Mueller M, Kramer D and Porter CW (1994) CGP 48664, a new *S*-adenosylmethionine decarboxylase inhibitor with broad spectrum antiproliferative and antitumor activity. *Cancer Res* **54:** 3210–3217

Seidenfeld J and Sprague WS (1990) Comparisons between sensitive and resistant human tumor cell lines regarding effects of polyamine depletion on chloroethylnitrosourea efficacy. *Cancer Res* **50:** 521–526

Seidenfeld J, Komar KA, Naujokas MF and Block AL (1986*a*) Reduced cytocidal efficacy for adriamycin in cultured human carcinoma cells depleted of polyamines by difluoromethylornithine treatment. *Cancer Res* **46:** 1155–1159

Seidenfeld J, Block AL, Komar KA and Naujokas MF (1986*b*) Altered cell cycle phase distributions in cultured human carcinoma cells partially depleted of polyamines by treatment with Difluoromethylornithine *Cancer Res* **46:** 47–53

Seidenfeld J, Barnes D, Block AL and Erickson LC (1987) Comparison of DNA interstrand cross-linking and strand breakage by 1,3-*bis* (2-chloroethyl)-1-nitrosourea in polyamine-depleted and control human carcinoma cells. *Cancer Res* **47:** 4538–4543

Sherman SE, Gibson D, Wang AHJ and Lippard SJ (1985) X-ray structure of the major adduct of the anticancer drug cisplatin with DNA: *cis*-[Pt($NH_3$)2{d(pGpG)}]. *Science* **230:** 412–417

Stjernborg L, Heby O, Mamont P and Persson L (1993) Polyamine-mediated regulation of *S*-adenosylmethionine decarboxylase expression in mammalian cells. *Eur J Biochem* **214:** 671–676

Thomas TJ and Messner RP (1988) Structural specificity of polyamines in lefthanded Z-DNA formation. *J Mol Biol* **201:** 463–467

Xiao L, Swank RA and Matthews HR (1991) Photoaffinity polyamines: sequence-specific interactions with DNA. *Nucleic Acids Res* **19:** 3701–3708

© Cancer Research Campaign 1997